# Lyme Endocarditis: A Case Report

**DOI:** 10.1093/ofid/ofaf623

**Published:** 2025-10-06

**Authors:** Mayyadah H Alabdely, Patricia Bartley, Carmela D Tan, Hannah Wang, Nabin K Shrestha

**Affiliations:** Department of Infectious Disease, Cleveland Clinic Foundation, Cleveland, Ohio, USA; Department of Infectious Disease, Cleveland Clinic Foundation, Cleveland, Ohio, USA; Department of Pathology and Laboratory Medicine, Cleveland Clinic, Cleveland, Ohio, USA; Department of Pathology and Laboratory Medicine, Cleveland Clinic, Cleveland, Ohio, USA; Department of Infectious Disease, Cleveland Clinic Foundation, Cleveland, Ohio, USA

**Keywords:** 16 seconds sequencing, *borrelia burgdorferi*, lyme endocarditis

## Abstract

A 67-year-old woman from Pennsylvania underwent mitral valve repair for regurgitation that developed shortly after Lyme disease treatment. Warthin-Starry stain revealed spirochetes in valve tissue and molecular testing found DNA of *Borrelia* spp. We report the first Lyme endocarditis case supported by visualization of spirochetes in cardiac valve tissue.

Lyme disease, caused by *Borrelia burgdorferi*, is the most common tick-borne infection in North America and Europe [1]. While early symptoms are often mild, later stages can involve the heart, nervous system, and joints [2]. Lyme carditis is an uncommon manifestation, typically presenting as conduction abnormalities, but in rare cases, it can progress to pancarditis or even endocarditis [3]. Lyme endocarditis is extremely rare, with only a few cases reported in the literature [4].

We report a clinically significant case of Lyme endocarditis with both molecular and histopathologic evidence of infection, including organisms visualized on Warthin-Starry stain consistent with spirochetes. This case provides more convincing evidence of this unusual manifestation of infection with this pathogen than prior reports, and highlights the value of a combined diagnostic approach.

## CASE REPORT

A 67-year-old woman from rural Pennsylvania with hypertrophic obstructive cardiomyopathy, hypertension, hyperlipidemia, and hypothyroidism, developed unexplained joint aches around November 2023, primarily affecting the knees, with some involvement of the shoulders. The patient was retired, lived with her husband in a wooded area, and enjoyed gardening and frequent walks outdoors, often in tick-infested environments. She did not smoke, she drank alcohol socially, and denied any illicit drug use. Her family history was notable for coronary artery disease in both parents, but there was no known history of autoimmune or rheumatologic disease. Although the patient lived in a tick-endemic area and had experienced numerous tick bites over the years, she had never found an engorged tick, and had never developed a target lesion in her skin. The patient's symptoms remained undiagnosed for several months until June 2024 when she requested Lyme disease testing from her primary care physician. Lyme antibodies and Western blot returned strongly positive. She was treated with a 10-day course of doxycycline, which resulted in substantial relief of her joint symptoms.

In September 2024, about three months after being initially diagnosed with Lyme disease, the patient began experiencing significant fatigue. In November 2024, she developed sudden-onset shortness of breath that worsened rapidly over several days. She presented to a local emergency department for evaluation. The patient had physical findings consistent with heart failure and echocardiography revealed the presence of severe mitral regurgitation. The patient was referred to our hospital for mitral valve replacement.

Transesophageal echocardiography revealed normal biventricular function, left ventricular outflow tract obstruction due to severe systolic anterior motion with interventricular septal thickening (∼1.4 cm), mild aortic regurgitation with a central jet, severe mitral regurgitation due to a perforation in the anterior mitral leaflet, and moderate tricuspid regurgitation due to annular dilation.

**Figure 1.**
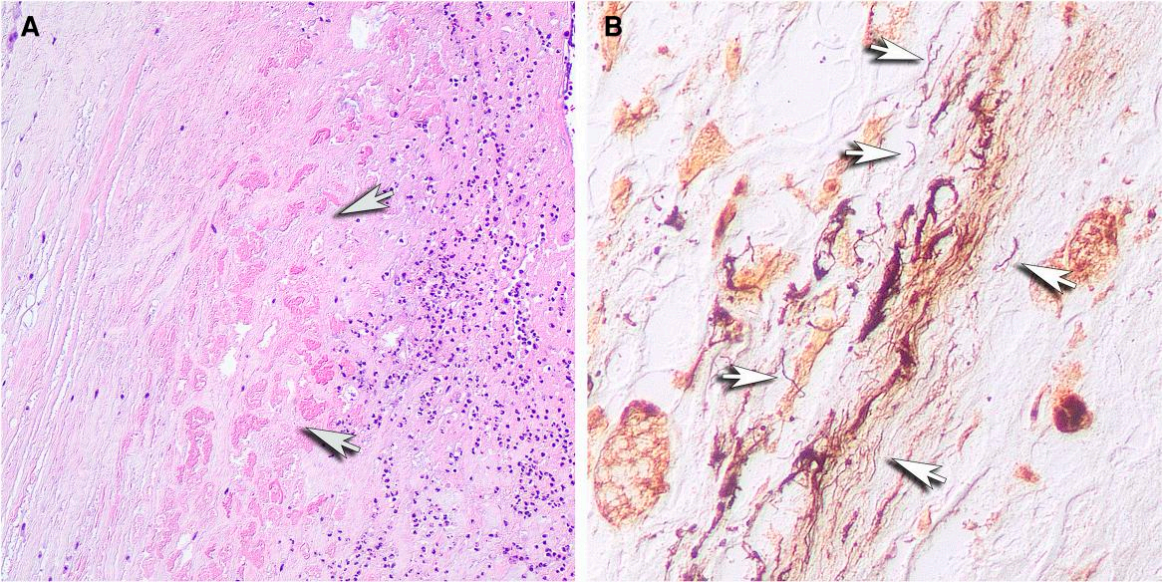
(*A*) Microscopy of the mitral valve showing acute and chronic inflammation with areas of necrobiosis. (*B*) Warthin-Starry stain of mitral valve tissue shows a focal cluster of filamentous bacterial organisms, with few organisms that are recognizable as spirochetes.

The patient subsequently underwent septal myectomy, complex mitral valve repair, and tricuspid valve repair. Histopathological examination of the excised mitral valve demonstrated the presence of areas of necrobiosis with acute inflammation and fibrinous vegetations. A Warthin-Starry silver stain revealed rare filamentous bacterial organisms consistent with spirochetes (Figure 1). Gram, periodic-acid Schiff (PAS), Gomori-methenamine silver (GMS), and Giemsa stains were negative for other organisms.

Broad range PCR and sequencing of the V1 to V3 region of the bacterial 16S rRNA gene was performed on fresh valve tissue and detected DNA of *Borrelia* species. Lyme serology was also positive, with all 10 diagnostic IgG bands detected (p93, p66, p58, p45, p41, p39, p30, p28, p23, and p18).

Based on the histopathological, molecular, and serological findings, and supported by a compatible clinical presentation, a diagnosis of Lyme endocarditis was made. The patient was treated with intravenous ceftriaxone and oral doxycycline for a total duration of 6 weeks. She completed this treatment without incident. When seen in follow-up after 6 months, she was doing well.

## DISCUSSION

Lyme carditis is a rare manifestation of Lyme disease, and it can involve various cardiac complications such as heart conduction abnormalities, myocarditis, pericarditis, endocarditis, pancarditis, arrhythmias, dilated cardiomyopathy, congestive heart failure, myocardial infarction, and coronary aneurysms [3, 4]. Among these, Lyme endocarditis is exceedingly rare, with only 9 previously reported cases in the literature [5–13].

Among the previously reported cases, only one case by Hidri et al. [7] demonstrated both histopathology and molecular confirmation of *Borrelia* species. In their case, the Warthin-Starry stain revealed scarce curved rods with a morphology not specific to spirochetes [7]. Rudenko et al. [10] reported a PCR-positive case; however, histopathology revealed only a highly calcified, dissected cardiac valve without demonstration of acute inflammation or microorganisms [10]. Haddad et al. [13] described another case with positive PCR results but negative histopathology [13]. In the case reported by Paim et al. [8] histopathology showed acute inflammation, but no organisms were visualized, although PCR was positive [8]. All the previous reports were included in a comprehensive review of published Lyme endocarditis cases from 1977 to July 2019 by Nikolić et al [4]. Since their review, only one additional report has been published. In this report, Gomez-Tschernko et al. [6] identified *Borrelia burgdorferi* through 16S rRNA PCR and sequencing of mitral valve tissue [6] (Table 1).

**Table 1.** Summary of Published Cases of Lyme Endocarditis

Author (Year)	Valve Involved	Serologic Testing	Histopathology Findings	Valve Culture	Molecular Testing	Treatment Given	Surgery Performed
Anish (1993) [5]	Aortic	EIA positive (1.10 → 1.22); Western blot IgG *P*-41 band positive	Not performed	Not performed	Not performed	Ceftriaxone IV × 2 wks → Doxycycline PO 100 mg BID × 30 d	No
Canver et al. (2000) [9]	Mitral	ELISA reactive (IgG & IgM); Immunoblot: 6 IgG bands	Myxoid degeneration with lymphocytic infiltration; no fibrinoid exudate or Aschoff bodies	Not performed	Not performed	Not specified	Yes
Rudenko et al. (2008) [10]	Aortic	ELISA reactive (IgG); Western blot positive	Highly calcified dissected valve	Negative for aerobic/anaerobic/spirochetes	PCR: 99% identity to *Borrelia bissettii* flagellin gene	Antimicrobial therapy unspecified	Yes
Hidri et al. (2012) [7]	Mitral	ELISA reactive (IgG); Immunoblot: 6 IgG bands	Foamy macrophages suggestive of intracellular organisms; Warthin-Starry: scarce curved rods; other stains negative	Not specified	16S rRNA PCR: *Borrelia* spp.; real-time PCR: *B. afzelii*	Valve replacement + IV gentamicin/amoxicillin × 2 wks → PO amoxicillin × 4 wks	Yes
Kameda et al. (2012) [11]	Mitral	ELISA reactive (IgG & IgM); Immunoblot positive	Not performed	Not performed	Not performed	Cefotaxime IV (200 mg/kg/day) × 14 d	No
Patel & Schachne (2017) [12]	Mitral	EIA positive: IgG (24.3), IgM (9.6), IgA (>9.9)	Not performed	Not performed	Not performed	Ceftriaxone IV → Doxycycline PO	No
Paim et al. (2018) [8]	Mitral	Negative for Bartonella, Coxiella, Chlamydia, Legionella, fungi, HIV	Active endocarditis; Gram, GMS, PAS-D, Steiner stains negative	Negative	16S rRNA PCR: *B. burgdorferi* from valve; blood PCR negative for *Borrelia* & *T. whipplei*	Ceftriaxone IV × 6 wks	Yes
Haddad et al. (2019) [13]	Mitral	EIA and Western blot positive (2 IgM, 10 IgG bands); other serologies negative	Limited tissue; non-diagnostic	Negative	16S rRNA sequencing of valve: *B. burgdorferi*	Ceftriaxone IV 2 g q24 h × 6 wks	Yes
Gomez-Tschernko et al. (2024) [6]	Mitral	Not reported	Not reported	Not reported	16S rRNA PCR and sequencing: *B. burgdorferi*	Surgical valve replacement + antimicrobial therapy	Yes

Our case provides stronger evidence of Lyme endocarditis than any of the prior published cases. In addition to demonstration of acute inflammation consistent with endocarditis on histopathological examination, Warthin-Starry stain demonstrated organisms morphologically consistent with spirochetes, broad range PCR and sequencing found DNA of *Borrelia* spp., and serology was strongly positive for *Borrelia burgdorferi*.

This case unequivocally demonstrates that Lyme endocarditis is a real clinical entity and underscores the importance of molecular and histopathological analysis in identifying the causative pathogen. Based on our current understanding that *Borrelia* can cause infective endocarditis, we suggest that in areas highly endemic for Lyme disease, PCR testing of blood for *Borrelia* could be considered in the workup of culture-negative endocarditis if there is no plan for cardiac surgery. For patients who undergo surgery, the pathogen will be identified by molecular microbiological testing of excised heart valve specimens.
